# Supplementary material for: Metabolic Regulation and Saline–Alkali Stress Response in Novel Symbionts of Epichloë bromicola-Bromus inermis
Source: Plants (Basel). 2025 Apr 1;14(7):1089. doi: 10.3390/plants14071089 (PMC11991420; doi:10.3390/plants14071089)
Supplement: Supplementary file 1 [file plants-14-01089-s001.zip › plants-3518231-supplementary.pdf]

Table S1. Sterile seedling inoculation method results.

Materials	Method	Fungi	Inoculation quantity	Survival quantity	Survival rate(%)	Number of infections	Infection rate(%)
Sterile seedling	Cut	*E.bromicola*	461	394	85.5	0	0
		E.guerinii	474	390	82.3	0	0
		E.elymi	470	385	84.0	0	0
		CK	472	392	83.1	0	0
	Slit	*E.bromicola*	1455	1136	78.1	24	2.1
		E.guerinii	462	370	80.1	0	0
		E.elymi	465	385	82.8	0	0
		CK	427	344	80.6	0	0
	Injection	*E.bromicola*	475	387	81.5	0	0
		E.guerinii	454	359	79.1	0	0
		E.elymi	467	396	84.8	0	0
		CK	417	347	83.2	0	0

Table S2. Seeds inoculation method results.

Materials	Method	Fungi	Inoculation quantity	Survival quantity	Survival rate(%)	Number of infections	Infection rate(%)
Seeds	Soaking	*E.bromicola*	500	371	74.2	0	0
		E.guerinii	500	360	72.0	0	0
		E.elymi	500	368	73.6	0	0
		CK	500	370	74.0	0	0
	Piercing and soaking	*E.bromicola*	500	325	65.0	3	0.9
		E.guerinii	500	344	68.8	0	0
		E.elymi	500	331	66.2	0	0
		CK	500	351	70.2	0	0
	Slit	*E.bromicola*	500	301	60.2	0	0
		E.guerinii	500	287	57.4	0	0
		E.elymi	500	261	52.2	0	0
		CK	500	304	60.8	0	0
